# Brain-derived neurotrophic factor regulates LYN kinase–mediated myosin light chain kinase activation to modulate nonmuscle myosin II activity in hippocampal neurons

**DOI:** 10.1016/j.jbc.2022.102054

**Published:** 2022-05-20

**Authors:** Xiaobing Li, Rong-Rong Yuan, Qixia Wang, Shouyu Chai, Zhengying Zhang, Yue Wang, Shu-Hong Huang

**Affiliations:** 1 Institute of Basic Medicine, Shandong Provincial Hospital Affiliated to Shandong First Medical University, Jinan, Shandong, China; 2 Institute of Basic Medicine, Shandong University, Jinan, Shandong, China; 3 Medical Science and Technology Innovation Center, Shandong First Medical University & Shandong Academy of Medical Sciences, Jinan, Shandong, China

**Keywords:** nonmuscle myosin II, brain-derived neurotrophic factor, LYN kinase, neuron, BDNF, brain-derived neurotrophic factor, co-IP, coimmunoprecipitation, DIV, days *in vitro*, IP, immunoprecipitation, LTP, long-term potentiation, MLC, myosin light chain, MLCK, MLC kinase, NM II, nonmuscle myosin II, ROCK, Rho kinase, SFK, Src family kinase, Trk, tyrosine kinase receptor, WB, Western blotting

## Abstract

Myosins belong to a large superfamily of actin-dependent molecular motors. Nonmuscle myosin II (NM II) is involved in the morphology and function of neurons, but little is known about how NM II activity is regulated. Brain-derived neurotrophic factor (BDNF) is a prevalent neurotrophic factor in the brain that encourages growth and differentiation of neurons and synapses. In this study, we report that BDNF upregulates the phosphorylation of myosin regulatory light chain (MLC2), to increases the activity of NM II. The role of BDNF on modulating the phosphorylation of MLC2 was validated by using Western blotting in primary cultured hippocampal neurons. This result was confirmed by injecting BDNF into the dorsal hippocampus of mice and detecting the phosphorylation level of MLC2 by Western blotting. We further perform coimmunoprecipitation assay to confirm that this process depends on the activation of the LYN kinase through binding with tyrosine kinase receptor B, the receptor of BDNF, in a kinase activity-dependent manner. LYN kinase subsequently phosphorylates MLCK, further promoting the phosphorylation of MLC2. Taken together, our results suggest a new molecular mechanism by which BDNF regulates MLC2 activity, which provides a new perspective for further understanding the functional regulation of NM II in the nervous system.

Myosins belong to a large superfamily of actin-dependent molecular motors. All myosins share a common functional domain––the motor domain––at the head of heavy chains, which has been shown to interact with actin, hydrolyze ATP, and produce movement (1). Phylogenetic analysis currently categorizes myosins into more than 15 classes. As a conventional myosin, myosin II has been extensively investigated. Myosin II was first discovered in muscle tissues, and it can couple ATPase activity, hydrolyze ATP to produce power, and mediate muscle contractions (2). Recently, myosin II has also been discovered in nonmuscle tissues, such as neurons; in these tissues, it is called nonmuscle myosin II (NM II). The expression level of NM II is high, and NM II plays an important role in the central nervous system. It is involved in neuron migration, neurite growth, axon guidance, microtubule transport in neuronal growth cones, actin dynamics, dendritic spine formation, and so on (3, 4, 5, 6, 7, 8, 9). Treatment with blebbistatin (a selective inhibitor of the ATPase activity of NM II) or myosin II RNAi resulted in loss of bulbous mushroom-like dendritic spine heads and an increase in filopodia-like protrusions in their place (10). Similarly, myosin II plays a role in advanced brain function. According to Rex *et al.* (6), myosin II is involved in the preservation of long-term potentiation (LTP), which is strongly implicated in memory. Myosin II expression and motor activity are also essential for long-term memory consolidation as well as the extinction of conditioned taste aversion. Induction of LTP can in turn induce myosin II activation, a process required for actin filament organization and stabilization in dendritic spine heads in rat hippocampal slices (6).

NM II consists of one pair of heavy chains and two pairs of light chains. Among the two pairs of light chains are one pair of regulatory chains and one pair of essential chains (11). Myosin light chain (MLC) kinase (MLCK) and Rho kinase (ROCK) modulate the activity of MLCs by phosphorylating MLC at Ser-19 or Ser-19/Thr-18. MLCK is Ca^++^ dependent (12).

In all of these reports, the researchers usually used shRNAs or inhibitors of heavy chains and ATPase activity to silence the function of myosin II. However, the manner in which myosin II participates in the nervous system remains unclear.

Brain-derived neurotrophic factor (BDNF) participates in the activities of cells as well as the brain, and it binds to two kinds of receptors––tyrosine kinase receptor (Trk) B and the low-affinity neurotrophic factor receptor (p75NTR)––to activate their downstream signaling pathways (13, 14, 15). In addition to promoting neuronal survival, differentiation, and neurite growth, BDNF also plays a key role in the regulation of synaptic plasticity (16). Synaptic plasticity is usually identified as the molecular basis of learning and memory, and it mediates information transfer between neurons. In cultured hippocampal neurons, BDNF can induce the production and maintain the stability of LTP. BDNF can also regulate actin dynamics to promote the formation of dendritic spines. Dendritic spines are specialized structures distributed on neuronal dendrites that can be involved in chemical signal transduction (13, 16, 17, 18, 19). However, even if BDNF regulates the activity of MN II’s light chain, the following two questions remain: first, which signaling pathway is involved in this regulation; second, how this regulation functions physiologically.

In this paper, we report that the BDNF/TrkB signaling pathway can upregulate the phosphorylation of regulatory MLC2 to increase the activity of NM II. We also demonstrate that this process does not depend on the three canonical signaling pathways downstream of TrkB; rather, it is mediated through LYN kinase in the Src family. This study contributes to an enhanced understanding of both the function and mechanism of NM II.

## Results

### BDNF promotes MLC2 phosphorylation through TrkB

**Figure 1:**
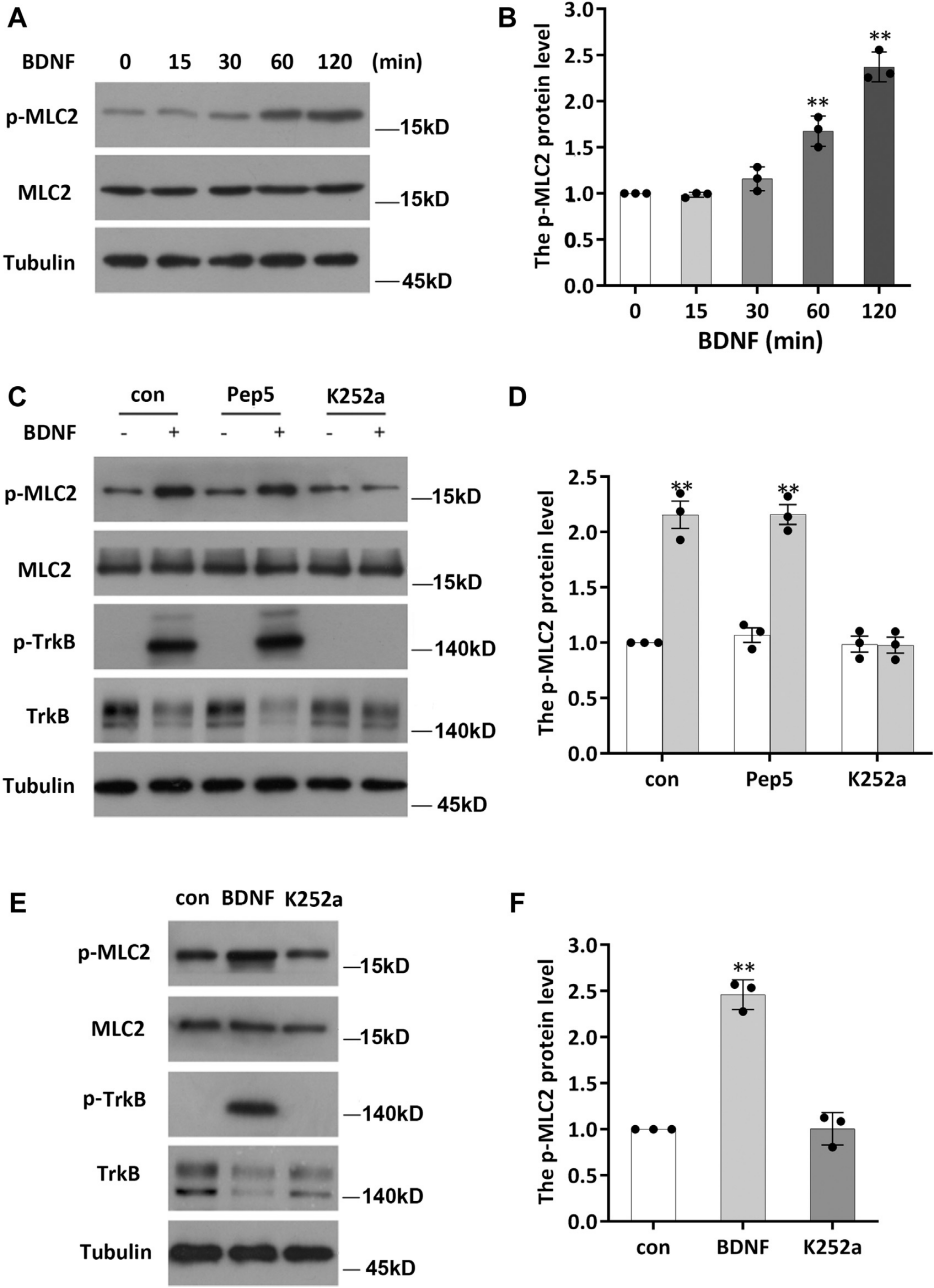
**BDNF promotes MLC2 phosphorylation through TrkB.***A*, starvation-treated (B27 removal from the medium for more than 6 h) DIV7 hippocampal neuronal cells were stimulated with 50 ng/ml BDNF at various timepoints, and the level of p-MLC2 was determined after cell lysis with TNE buffer. The experiment was repeated three independent times. *B*, statistical results of p-MLC2 changes at different times during BDNF stimulation; the data are shown as the mean ± S.E.M. values; one-way ANOVA, ∗∗*p* < 0.01. *C*, DIV7 hippocampal neurons subjected to starvation were stimulated with BDNF (50 ng/ml) for 2 h before the addition of K252a (10 μM) or p75NTR peptide (10 μM) for 30 min, and the levels of p-TrkB and p-MLC2 were determined after lysis of the cells with TNE buffer. The experiment was performed with three independent replicates. *D*, the level of p-MLC2 was determined after 2 h of BDNF stimulation following pretreatment with pep5 or K252a 30 min; the data are shown as the mean ± S.E.M. values, Student’s *t* test, ∗∗*p* < 0.01. *E* and *F*, the levels of p-MLC2 and MLC2 were measured in the dorsal hippocampus 2 h after injection of K252a or BDNF. The experiment was performed with three independent replicates. The data are shown as the mean ± S.E.M. values; one-way ANOVA, ∗∗*p* < 0.01. BDNF, brain-derived neurotrophic factor; DIV, days *in vitro*; MLC, myosin light chain; TrkB, tyrosine kinase receptor B.

To examine whether BDNF can modulate the activity of NM II, we assessed the phosphorylation level of MLC2 in primary cultured rat hippocampal neurons upon BDNF treatment. Hippocampal neurons were cultured for 7 days (days *in vitro* [DIV]7), and B27 was then removed over a 6 h period. This step was followed by BDNF (50 ng/ml) treatment for various time periods. Cell lysates were then collected at different times. NM II activation was monitored by immunoblotting with phospho-MLC2–specific antibodies, and the ratio of p-MLC2 to MLC2 was calculated. As shown in Figure 1*A*, we found that the phosphorylation level of MLC2 began to increase 30 min after neurons were stimulated with BDNF and continued to increase with time (Fig. 1, *A* and *B*). BDNF can bind to two receptors on the cell surface with different affinity (the high-affinity TrkB and the low-affinity p75 receptor [p75NTR], triggering different downstream signaling responses. To further confirm which receptor is involved in the regulation of MLC2 phosphorylation by BDNF, we separately treated DIV7 neurons with two receptor inhibitors—K252a (a Trk inhibitor, 100 nM, a concentration that can inhibit Trk activity) and tat-pep5 (a p75NTR inhibitor, 100 nM)—30 min prior to BDNF treatment to inhibit the two signaling pathways (20). Then, the neurons were stimulated with BDNF for 2 h. The levels of p-MLC2 and MLC2 were determined by Western blotting (WB). We found that pretreatment with K252a inhibited activation of TrkB and blocked MLC2 phosphorylation triggered by BDNF, while the addition of tat-pep5 did not affect the response of MLC2 to BDNF (Fig. 1, *C* and *D*). To verify the results *in vivo*, we injected K252a or BDNF into the dorsal hippocampus of mice and found that the level of p-MLC2 increased after injection of BDNF, whereas the addition of K252a decreased the level of p-MLC2 (Fig. 1, *E* and *F*). From these *in vivo* and *in vitro* experiments, we found that BDNF can modulate NM II activity by regulating MLC2 phosphorylation and that this modulation depends on TrkB.

### BDNF regulates MLC2 phosphorylation by activating MLCK

**Figure 2:**
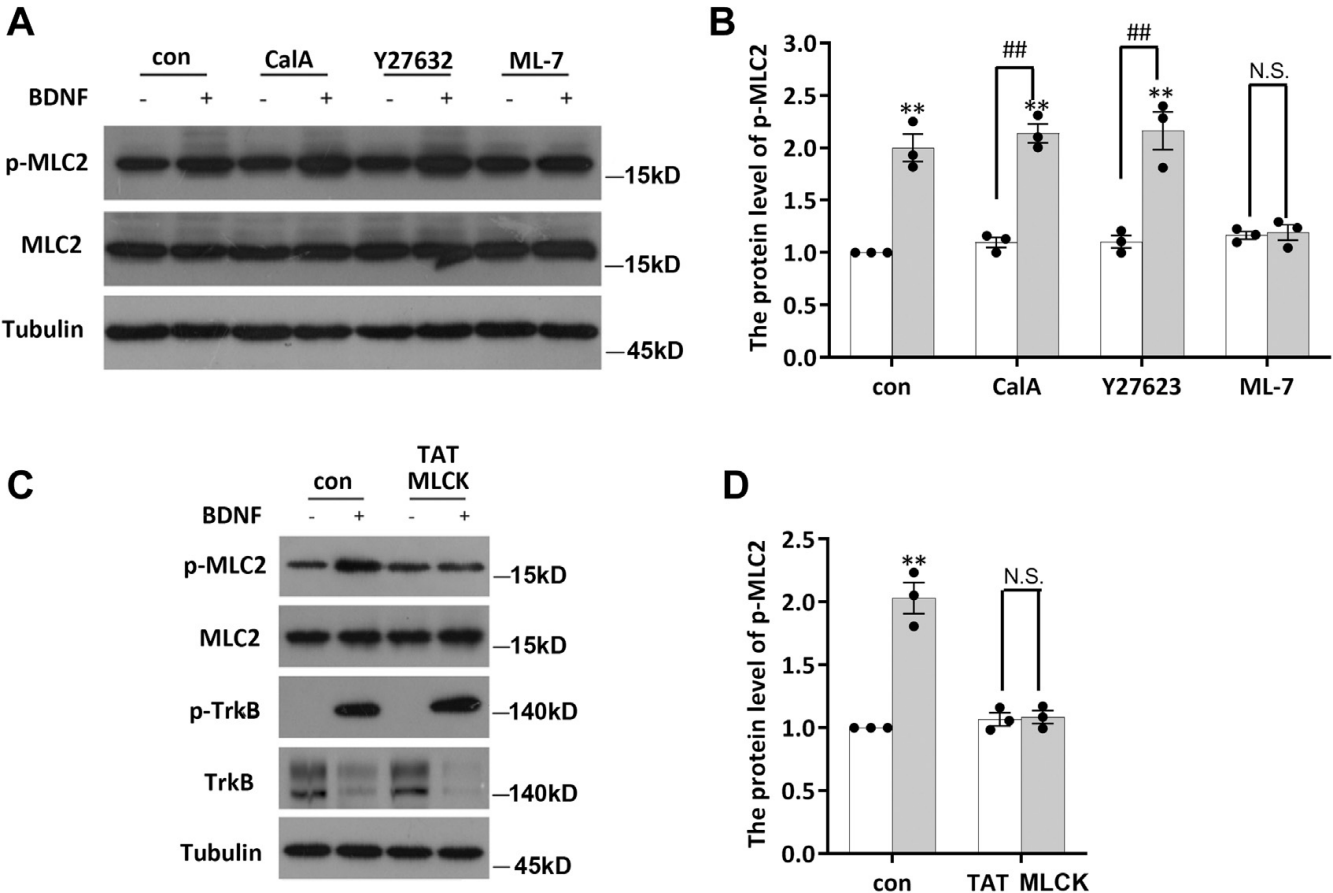
**BDNF regulates MLC2 phosphorylation by activating MLCK.***A*, DIV7 hippocampal neurons were stimulated with BDNF (50 ng/ml) for 2 h after the addition of calyculin A (10 μM), ML-7 (10 μM), or Y27623 (15 μM) for 30 min, and cells were lysed to collect protein for the measurement of p-MLC2 and total MLC2 levels. The experiment was performed with three independent replicates. *B*, statistical results of (*A*); the data are shown as the mean ± S.E.M. values; Student’s *t* test, ∗*p* < 0.05. *C*, the levels of MLCK, MLC2, and p-MLC2 were measured after 2 h of BDNF (50 ng/ml) stimulation after blocking the binding of MLCK to MLC2 by treatment with the specific peptide TAT-MLCK. The assay was performed with three independent replicates. *D*, statistical results of (*C*); the data are shown as the mean ± S.E.M. of three independent replicates; Student’s *t* test, ∗*p* < 0.05. BDNF, brain-derived neurotrophic factor; DIV, days *in vitro*; MLC, myosin light chain; MLCK, MLC kinase.

The phosphorylation of MLC2 is mainly regulated by two kinases, MLCK and ROCK (12, 21), and it can be caused by a decrease in protein phosphatase activity (22). Therefore, we sought to further verify which kinase or phosphatase is primarily involved in the phosphorylation of MLC2 induced by BDNF/TrkB. We pretreated serum-starved hippocampal neurons with calyculin A (a protein phosphatase inhibitor, 10 μM) to prolong MLC2 phosphorylation and activity, ML-7 (an MLCK inhibitor, 10 μM) (23, 24), or Y-27623 (a ROCK inhibitor, 15 μM) (25). We added the inhibitors 30 min before BDNF treatment. Afterward, hippocampal neurons were stimulated with BDNF for 2 h (in the presence of the inhibitors). The inhibitor working concentrations and treatment times were chosen according to a previous reference. We then lysed cells, collected protein, and performed WB to measure the levels of the corresponding proteins. When ML-7 was added to inhibit the activity of MLCK, the phosphorylation level of MLC2 did not increase upon stimulation with BDNF, whereas the ROCK inhibitor and the protein phosphatase inhibitor did not affect this process (Fig. 2, *A* and *B*). To confirm this result, we synthesized peptides to specifically block the binding of MLCK and MLC2 (TAT-MLCK). When the TAT peptides were added to the medium, MLC2 no longer responded to BDNF (Fig. 2, *C* and *D*). These results suggest that BDNF regulates the phosphorylation of MLC2 by activating MLCK.

### Src signaling is involved in the regulation of MLC2

**Figure 3:**
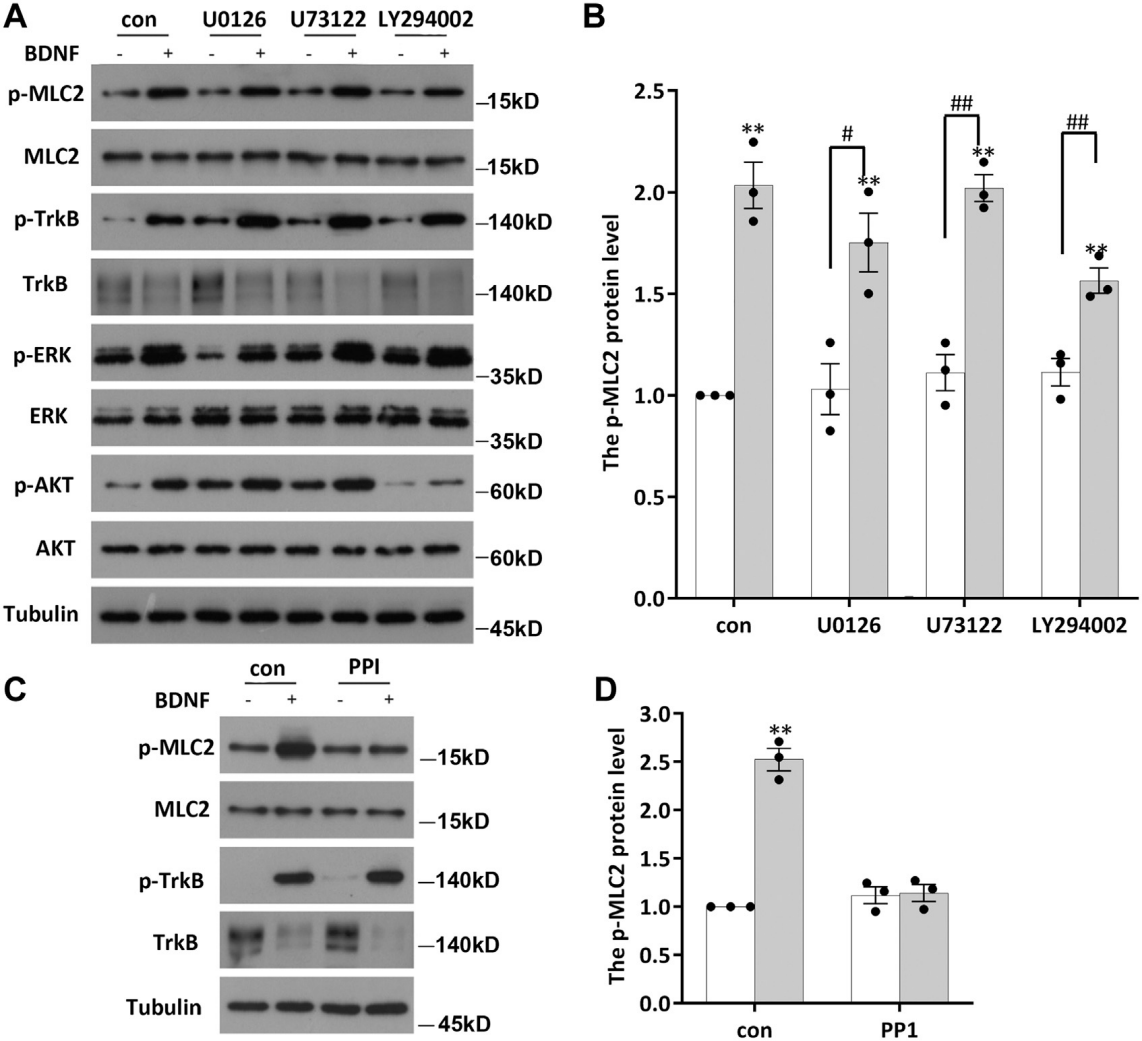
**SRC signaling is involved in the regulation of MLC2 by BDNF.***A*, DIV7 neurons subjected to starvation were pretreated with inhibitors of three established signaling pathways downstream of TrkB: U0126 (a MAPK signaling pathway inhibitor, 10 μM), U73122 (a PLC-gamma signaling pathway inhibitor, 10 μM), and LY294002 (a PI3K signaling pathway inhibitor, 10 μM) for 30 min. This treatment was followed by BDNF treatment (50 ng/ml) for 2 h, and cells were then lysed to collect proteins to examine the activity of the three signaling pathways downstream of TrkB as well as the levels of MLC2 and p-MLC2. The experiment was performed in triplicate. *B*, data are shown as the mean ± S.E.M. of three independent replicates; Student’s *t* test, ∗*p*< 0.05. *C* and *D*, after being pretreated for 30 min with PP1 (10 μM), cells were stimulated with BDNF (50 ng/ml) for 2 h and lysed, and the protein levels of MLC2 and p-MLC2 were determined by Western blotting. The assay was performed with three independent replicates. The data are shown as the mean ± S.E.M. values; Student’s *t* test, of three independent replicates, ∗*p* < 0.05. BDNF, brain-derived neurotrophic factor; DIV, days *in vitro*; MLC, myosin light chain.

After BDNF binds to TrkB, TrkB is dimerized and autophosphorylated to activate the three canonical signaling pathways MAPK, PI3K, and PLC-gamma, which thereby perform their corresponding functions (26). To address the question of which signaling pathway is involved in BDNF-triggered phosphorylation of MLC2, DIV7 neurons were pretreated with U0126 (10 μM, a MAPK signaling pathway inhibitor), U73122 (10 μM, a PLC-gamma signaling pathway inhibitor), or LY294002 (10 μM, a PI3K signaling pathway inhibitor) for 30 min and were then stimulated with BDNF for 2 h (in the presence of the inhibitors). The inhibitor working concentrations and treatment times were chosen according to a previous reference. We subsequently detected the phosphorylation of MLC2 by WB. As shown in Figure 3, *A* and *B*, we found that the phosphorylation level of MLC2 did not change significantly compared with that in the control group after pretreatment with the three signaling pathway inhibitors.

**Figure 4:**
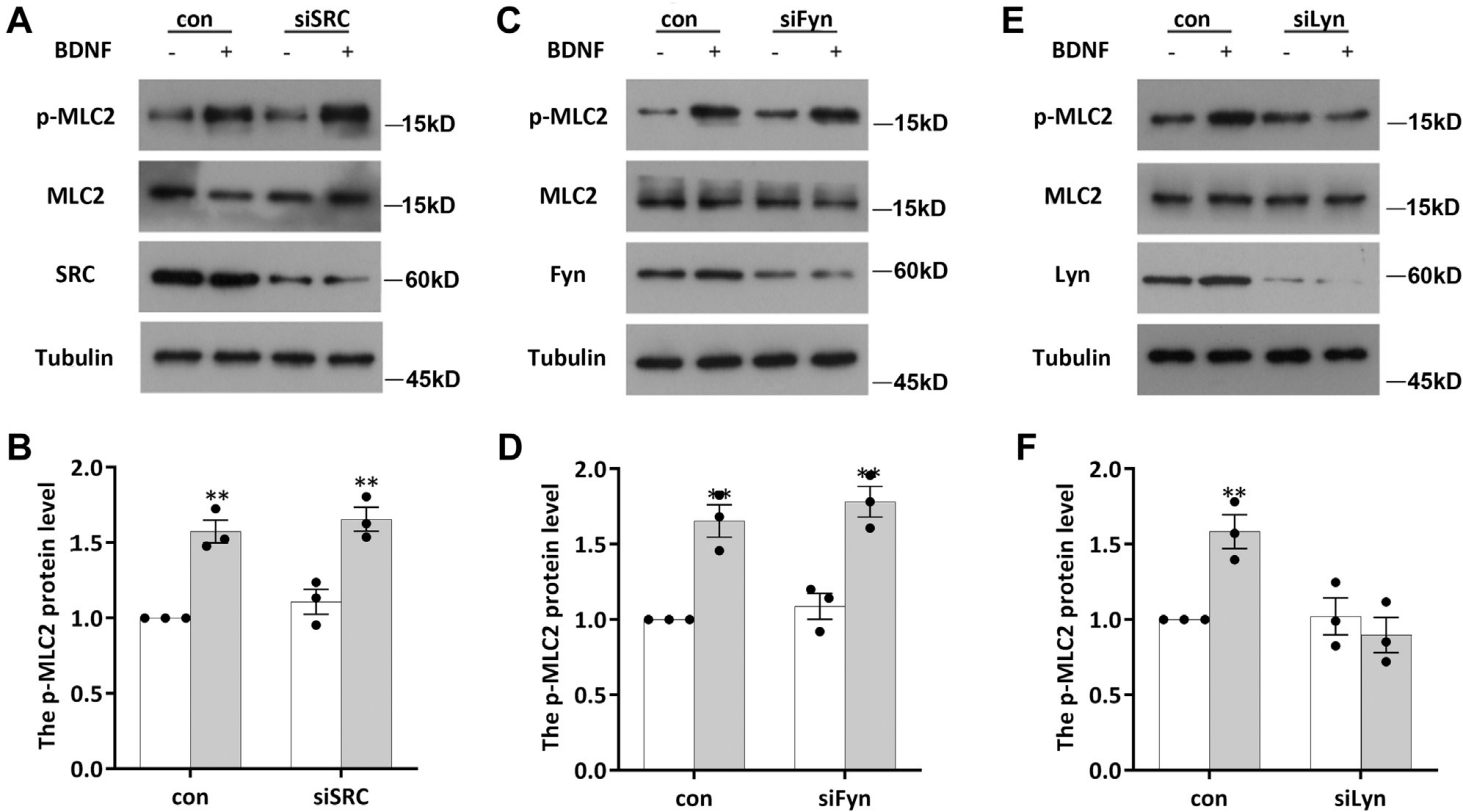
**LYN kinase is involved in the regulation of MLC2 phosphorylation by BDNF.***A* and *B*, DIV7 neurons were transfected with siSRC, siFYN, or siLYN for 48 h prior to 2 h of BDNF stimulation, and the levels of MLC2 and p-MLC2 were determined by Western blotting. The experiment was performed with three independent replicates. The data are shown as the mean ± S.E.M. values; Student’s *t* test, of three independent replicates, ∗*p* < 0.05. *C* and *D*, DIV7 neurons were transfected with siFYN for 48 h prior to 2 h of BDNF stimulation, and the levels of MLC2 and p-MLC2 were determined by Western blotting. The experiment was performed with three independent replicates. The data are shown as the mean ± S.E.M. values; student’s *t* test of three independent replicates, ∗*p* < 0.05. *E* and *F*, DIV7 neurons were transfected with siLYN for 48 h prior to 2 h of BDNF stimulation, and the levels of MLC2 and p-MLC2 were determined by Western blotting. The experiment was performed with three independent replicates. The data are shown as the mean ± S.E.M. values; student’s *t* test of three independent replicates, ∗*p* < 0.05. BDNF, brain-derived neurotrophic factor; DIV, days *in vitro*; MLC, myosin light chain.

**Figure 5:**
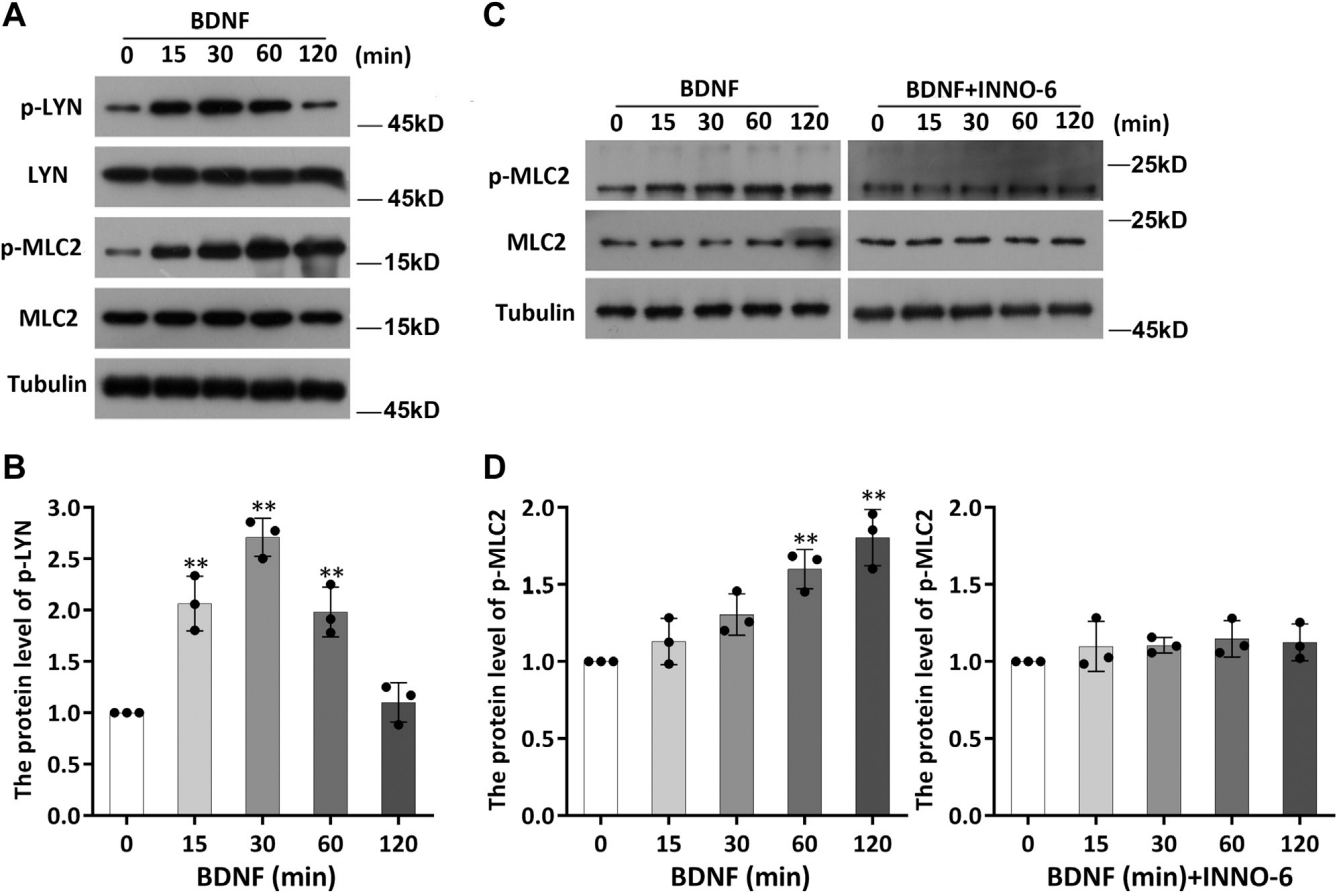
**Blocking LYN activity ensures that BDNF no longer regulates MLC2 phosphorylation changes.***A* and *B*, DIV7 neurons subjected to starvation were stimulated with BDNF (50 ng/ml) for various times, and the levels of LYN and p-LYN were determined by Western blot analysis. The experiment was performed with three independent replicates. The data are shown as the mean ± S.E.M. values; one-way ANOVA of three independent replicates, ∗*p*< 0.05. *C* and *D*, DIV7 neurons subjected to starvation were stimulated for various times with bafetinib, a specific inhibitor of LYN, and the levels of MLC2 and p-MLC2 were determined by Western blot analysis. The experiment was performed with three independent replicates. The data are shown as the mean ± S.E.M. values; one-way ANOVA of three independent replicates, ∗*p* < 0.05. BDNF, brain-derived neurotrophic factor; DIV, days *in vitro*; MLC, myosin light chain; MLCK, MLC kinase.

BDNF increases the activity of Src family kinases (SFKs) following TrkB activation (27, 28). SFKs are nonreceptor tyrosine kinases that are involved in the regulation of many important cellular functions (29). This observation prompts the question of whether SFKs are involved in the phosphorylation of MLC2 induced by BDNF. DIV7 neurons were pretreated with PP1 (10 μM, a highly selective Src inhibitor) for 30 min before BDNF stimulation, and the resulting p-MLC2 levels were measured by WB (Fig. 3, *C* and *D*). We found that the addition of PP1 did not affect the phosphorylation level of MLC2 compared to that in the control group under basal conditions; however, after PP1 pretreatment, BDNF no longer upregulated MLC2 phosphorylation (Fig. 3, *C* and *D*). These results suggest that SFKs are involved in the phosphorylation of MLC2 triggered by BDNF. In vertebrates, the Src kinase family is composed of nine members: SRC, LCK, LYN, BLK, HCK, FYN, FGR, YES, and YRK. Among them, FYN, SRC, and LYN are distributed throughout the brain (30, 31). To further the kinase(s) involved in this process, we transfected siRNA for each kinase into neurons for 48 h to interfere with their expression. Neurons were then stimulated with BDNF for 2 h, and the p-MLC2 level in each group was determined. We found that knockdown of FYN and SRC did not affect the response of MLC2 to BDNF (Fig. 4, *A*–*D*). However, when the expression of LYN was knocked down by siRNA, BDNF no longer triggered an increase in the p-MLC2 level (Fig. 4, *E* and *F*). This result suggests that LYN is involved in BDNF-induced MLC2 phosphorylation. To further confirm the involvement of LYN in this process, DIV7 neurons were stimulated with BDNF for various times, and the phosphorylation level of LYN was determined. We found that the p-LYN level began to increase significantly 30 min after BDNF addition and returned to the basal level by 2 h (Fig. 5, *A* and *B*). We further pretreated neurons with bafetinib (a Lyn-specific inhibitor) for 30 min and then stimulated them with BDNF for various times to detect p-MLC2. The results demonstrated that the phosphorylation of MLC2 was no longer regulated by BDNF after treatment with bafetinib to specifically inhibit LYN activity (Fig. 5, *C* and *D*). Taken together, the aforementioned results show that BDNF activates the downstream LYN kinase to participate in the regulation of MLC2 phosphorylation after binding to TrkB.

### LYN interacts with TrkB

**Figure 6:**
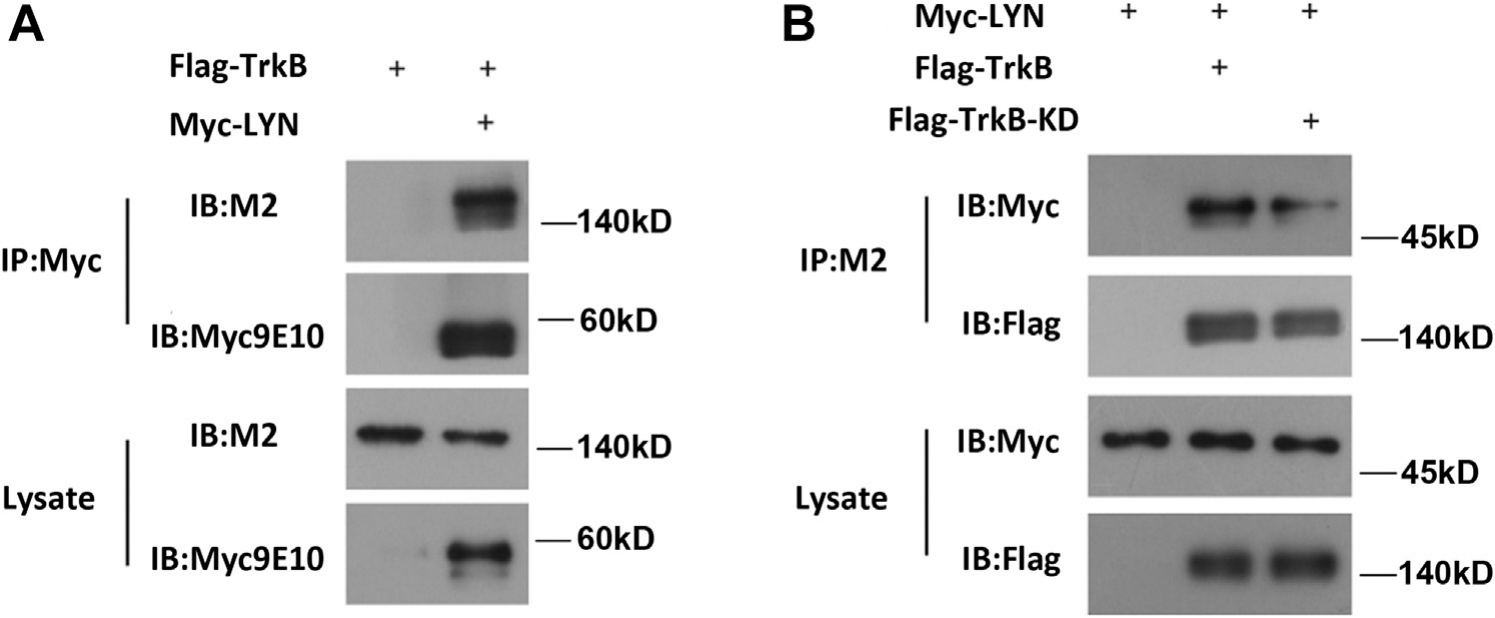
**LYN interacts with TrkB.***A*, HEK293 cells were transfected with LYN-Myc and FLAG-TrkB, and the binding of LYN to TrkB was assessed by coimmunoprecipitation. An anti-Myc antibody (9E10) was used for IP, and rabbit anti-FLAG and anti-Myc antibodies were used for Western blotting. The assay was performed with three independent replicates. *B*, HEK293 cells were transfected with LYN-Myc (CON group), FLAG-TrkB, or FLAG-TrkB-KD, and the binding of LYN to TrkB was assessed by coimmunoprecipitation. An anti-FLAG antibody (M2) was used for IP, and rabbit anti-FLAG and anti-Myc antibodies were used for Western blotting. The assay was performed with three independent replicates. TrkB, tyrosine kinase receptor B.

Furthermore, we sought to investigate whether LYN binds to TrkB. We generated constructs expressing Myc-tagged LYN, FLAG-tagged TrkBwt, and a FLAG-tagged TrkB–kinase-dead mutant (FLAG-TrkB-KD). We then overexpressed LYN-Myc, FLAG-TrkB, and FLAG-TrkB-KD in the HEK 293 cell line. Binding was detected by coimmunoprecipitation (co-IP). The anti-FLAG antibody M2 and an anti-Myc antibody were used for immunoprecipitation (IP). The results showed that LYN can bind to TrkB. After eliminating the kinase active site in TrkB (TrkB-KD), the binding rate of LYN to TrkB decreased significantly. The results suggest that the binding of Lyn to TrkB depends on the kinase activity of TrkB (Fig. 6, *A* and *B*).

### LYN acts as an upstream kinase to regulate MLCK phosphorylation

**Figure 7:**
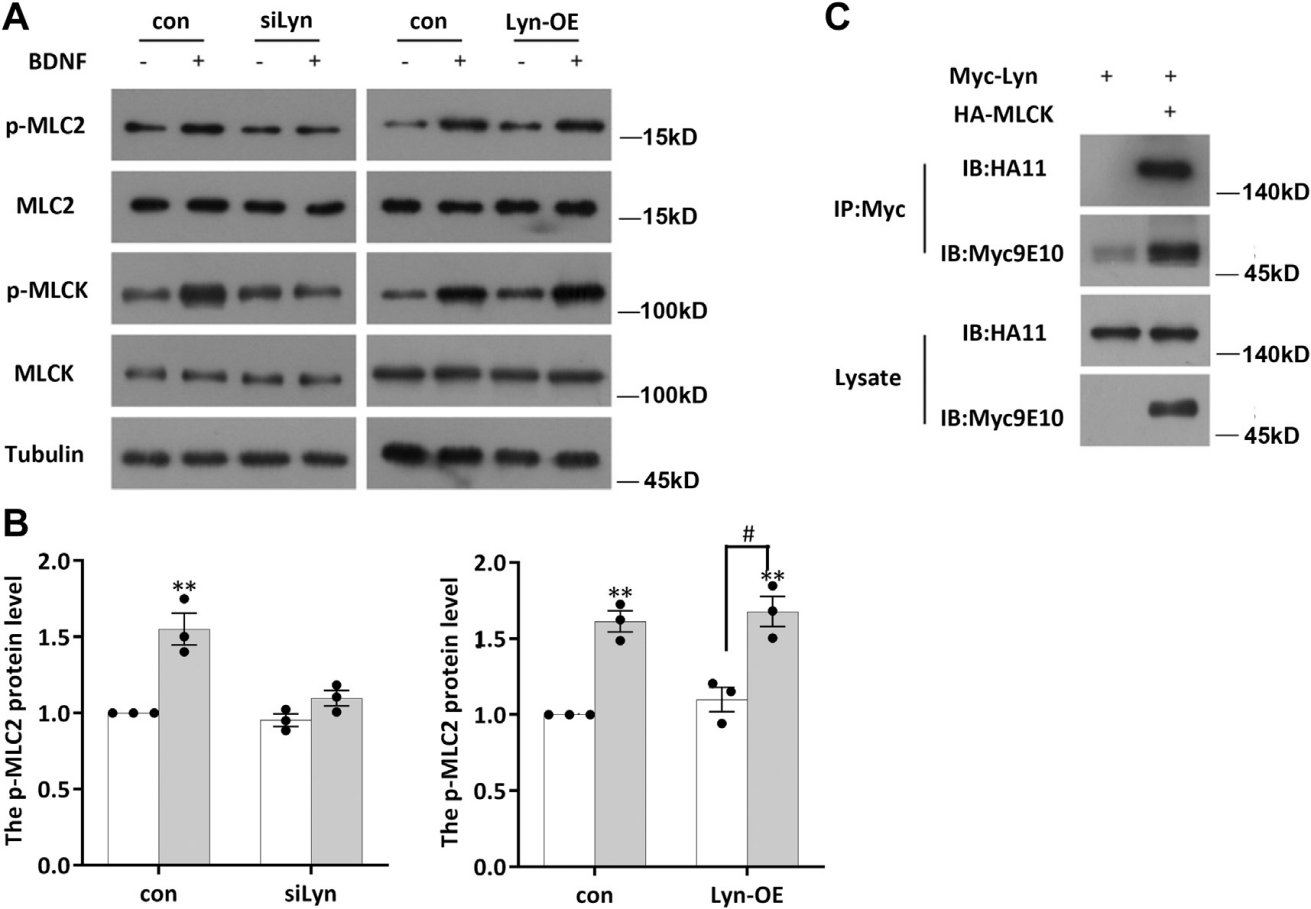
**LYN regulates MLCK phosphorylation.***A*, DIV7 neurons subjected to starvation were transfected with siLYN to interfere with LYN expression (*left*) or with the LYN-Myc overexpression plasmid (*right*), and the protein levels of MLCK, P-MLCK, MLC2, and p-MLC2 were determined by Western blotting. The experiment was performed with three independent replicates. *B*, data are shown as the mean ± S.E.M. values; student’s *t* test of three independent replicates, ∗*p* < 0.05. *C*, Lyn-Myc and MLCK-HA were overexpressed in HEK293 cells, and the binding of LYN to MLCK was assessed by IP using anti-HA and anti–c-*myc* antibodies, respectively. Three independent replicates of this experiment were performed. DIV, days *in vitro*; MLC, myosin light chain; MLCK, MLC kinase.

Next, we sought to address whether LYN kinase regulates MLCK activity directly. SRC can rapidly phosphorylate MLCK and increase its enzymatic activity by twofold to threefold *in vitro*. We transfected siRNA (si-LYN) into PC12 cells (a neuron-like cell line) to stably express TrkB for 72 h, and we then starved the cells overnight, stimulated them with BDNF for 2 h, and determined the phosphorylation levels of MLCK and MLC2 by WB. The results showed that MLCK phosphorylation decreased significantly after knockdown of LYN; moreover, MLC2 no longer responded to BDNF, and the level of p-MLC2 remained unchanged (Fig. 7, *A* and *B*). We also sought to determine whether LYN kinase can interact with MLCK. We generated constructs expressing Myc-tagged LYN and HA-tagged MLCK. In HEK293 cells transfected with those constructs, we determined whether LYN and MLCK had a physiological interaction by co-IP. As shown in Figure 7*C*, HA-MLCK was pulled down by Myc-LYN. This finding suggests that MLCK is a downstream target of LYN.

## Discussion

NM II is an important intracellular motor protein. It has been reported that NM II is involved in the migration of central neurons, the growth and guidance of axon processes, the state and transport of microtubules in the growth cone of neurons, actin dynamics, the formation of dendritic spines, and the maintenance of normal neuronal morphology (3, 7, 8, 11, 32, 33, 34, 35). NM II is also widely involved in higher functions of the brain, including learning and memory.

BDNF is a neurotrophic factor that is widely expressed in the brain (13, 16, 17, 18, 19). There is substantial overlap between the functions of BDNF and NM II, such as the migration of central neurons, the growth and guidance of axon processes, and the formation of memory. Little is known about any potential coordination between BDNF and NM II.

In this paper, we report that BDNF/TrkB signaling pathway activity can upregulate the phosphorylation of regulatory MLC2 to increase MN II activity. Furthermore, we showed that this process depends on the LYN signaling pathways downstream of TrkB. The findings of this study can facilitate an improved understanding of both the function and mechanism of action of NM II.

Our results provide several new insights into the mechanism by which the activity of NM II is controlled. First, we demonstrated that BDNF can increase the level of p-MLC2 in primary cultured hippocampal neurons. It was further shown that this process depends on the high affinity receptor TrkB. Furthermore, we showed that the same phenomenon occurs in the brains of mice. We injected normal saline into the dorsal hippocampus in the control group and injected BDNF or K252a + BDNF into the dorsal hippocampus in the experimental groups. The *in vivo* results were consistent with the *in vitro* results, meaning that BDNF could promote the phosphorylation of MLC2 through TrkB in the brain. Our study found for the first time that BDNF regulates the activity of MLC2 at both the cellular and systemic levels.

Second, we reported that BDNF is dependent on LYN in the Src family to promote the phosphorylation of MLC2. We found that inhibitors of the three traditional TrkB signaling pathways (U0126, LY294002, and U73122) did not affect the level of p-MLC2 in cultured neurons and that the MLC2 phosphorylation level remained increased after BDNF stimulation. Next, we found that TrkB can upregulate the phosphorylation of MLC2 by activating SFK signaling. By treating neurons with a broad-spectrum SRC signaling inhibitor and siRNAs for different Src family members, we discovered that treatment with the SRC inhibitor PP1 or LYN siRNA inhibited the BDNF-induced changes in p-MLC2. Bafetinib, a specific inhibitor of LYN kinase, blocked the response of LYN to BDNF. Bafetinib is an antitumor drug used in clinical medicine. Our study provides important insights for further study of its physiological function. We demonstrated by co-IP assays that TrkB interacts with LYN and that this binding depends on the activity of TrkB. It is suggested that TrkB may activate LYN through direct binding, which is similar to the mechanism by which EGFR activates LYN.

Third, we verified that LYN upregulates the phosphorylation of MLC2 by activating MLCK. SRC kinase can promote MLCK activity by directly binding to MLCK. We further demonstrated that LYN also binds to MLCK and that reducing the expression of LYN can inhibit MLCK activity, whereas exogenous overexpression of LYN can enhance MLCK activity.

As mentioned, neuronal structure is stabilized by the cytoskeleton. The molecular motor protein (NM II) binds to actin filaments, whose contractile properties further endow the cytoskeletal network with highly dynamic control of neuron behavior and architecture. However, the upstream regulatory mechanism of NM II is not well known. Our study elucidated the regulatory network connecting BDNF/TrkB signaling, Lyn kinase signaling, and NM II. Because both BDNF/TrkB signaling and Lyn kinase have a variety of inhibitors used in clinical treatment, such as belizatinib and bafetinib, we should consider the effect of NM II activity and nervous system injury caused by related effects when using these drugs in the clinic. Targeted strategies to protect the activity of NM II in neurons have potential clinical significance.

## Conclusions

Based on the aforementioned results, we confirmed that BDNF is involved in the regulation of MLC2 phosphorylation, which occurs *via* activation of the SFK LYN downstream of TrkB and then *via* activation of MLCK. We identified the molecular mechanism by which BDNF regulates MLC2 activity, and this finding provides a new perspective for further understanding the functional regulation of NM II in the nervous system.

## Experimental procedures

### Chemicals and antibodies

ML-7 HCl (S8388), Y-27623 (S6390), U0126 (S1102), U73122 (S8011), LY294002 (S1105), PPI (S7060), and bafetinib (INNO-406) were purchased from Selleck (Selleck Chemicals). The following antibodies were purchased: rabbit anti-TrkB (#07-225) from Millipore; mouse anti-FLAG (M2), rabbit anti-HA, mouse IgG Sepharose, and mouse antitubulin from Sigma–Aldrich; rabbit anti-FLAG from Thermo Fisher Scientific; mouse or rabbit anti-p-MLCK and mouse anti–c-*myc* (9E10) from Santa Cruz Biotechnology; rabbit anti–c-*myc* from Bethyl Laboratories; rabbit anti-MYL9 from ProteinTech; mouse anti-HA (HA.11) from Covance Research Products; and rabbit anti-p44/42 MAP kinase, mouse antiphospho-p44/42 (Erk1/2; Thr202/Tyr204), rabbit antiphospho-TrkA (pY490), and horseradish peroxidase–conjugated goat antimouse or rabbit IgG from Calbiochem. Human recombinant BDNF was obtained from PeproTech. Restriction enzymes were purchased from Ferments. Mounting medium was obtained from Vector Laboratories (H-1000). The other reagents were obtained from Sigma–Aldrich.

### Cell culture and transfection

Hippocampal neurons from pregnant Sprague–Dawley rats (E18) were cultured as previously described (Huang *et al.*, 2011). In brief, embryos were removed from rats on embryonic day 18 and were then maintained in Hanks’ balanced salt solution without Ca^2+^ and Mg^2+^. Hippocampal tissue was isolated from embryos, digested with 0.05% trypsin-EDTA at 37 °C for 5 min, and then mechanically ground and separated in a 1:1 mixture of Dulbecco's modified Eagle's medium (DMEM)/F-12 (Life) and 10% fetal bovine serum with a sterile, flame-polished glass Pasteur pipette. Neurons were cultured on a cover glass coated with 0.1 mg/ml poly-D-lysine (Sigma–Aldrich) in a 6-well plate in neural basic medium supplemented with 2% B27 and 0.5 mM glutamine. Cells were incubated in an incubator under conditions of saturated humidity, 5% CO_2_, and a constant temperature of 37 °C. Neurons cultured *in vitro* for 3 days (DIV3) were transferred to Opti–MEM (Invitrogen) using Lipofectamine 3000 transfection reagent (Invitrogen) according to the manufacturer's instructions. HEK293 cells were cultured in high-glucose DMEM (Invitrogen) supplemented with 10% fetal bovine serum (Life), 100 U/ml penicillin, and 100 μg/ml streptomycin. Crosscontamination with other human cell lines was evaluated, and all cells tested negative. siRNA and overexpression plasmids were transfected using Lipofectamine RNAiMAX (#13778500) and Lipofectamine 2000 (#11668019, Invitrogen), respectively, according to the manufacturer’s instructions.

### Plasmid constructs

The rat TrkB-FL, FLAG-rTrkB, and TrkB-KD constructs were prepared as described previously. Myc-tagged human LYN and HA-tagged human MLCK were subcloned into the pCDNA3.1 expression plasmid.

### Co-IP assay

Forty-eight hours after being electroporated with the indicated constructs with a Lonza Amaxa Biosystems electroporator, HEK293 cells were extracted with IP buffer (10 mM Tris [pH 8.0], 150 mM NaCl, 1 mM EDTA, 1% NP-40, and 10% glycerol with protease inhibitors). Cell lysates were clarified by centrifugation at 14,000*g* for 15 min at 4 °C. After centrifugation, the soluble supernatants were incubated with the indicated antibodies for 4 h at 4 °C. Immunocomplexes were then precipitated with protein A-Sepharose or protein G-Sepharose (Sigma) overnight at 4 °C. The beads were then washed three times with IP buffer, eluted by boiling in SDS–PAGE sample buffer, and finally analyzed by immunoblotting with the indicated antibodies.

### Surgery and microinjection

The mice were cared for in strict accordance with the institutional guidelines for animal care and the experiment was approved by the Committee on the Ethics of Animal Care and Use, Institute of Basic Medicine, Shandong Academy of Medical Sciences. Before surgery, mice were anesthetized with 5% chloral hydrate (0.8 ml/100 g, i.p.). Agents were injected into the dorsal hippocampus of mice using a Hamilton microsyringe. The coordinates (in reference to the bregma) were as follows: dorsal hippocampus—anteroposterior(AP), 1.7 mm; lateral (L), 1.5 mm; dorsoventral (V), 2.3 mm. The infusions were performed in a volume of 1 μl over 2 min, and the infusion cannula was kept in place for diffusion for an additional 4 min. Mice were killed 2 h after microinjection. This research study was approved by the Institutional Review Board of Shandong First Medical University.

### Statistical analysis

Data were obtained from three separate experiments. Experimental data were analyzed with SPSS software (IBM). The statistical methods used were one-way ANOVA, Student's *t* test, and the chi square test. All statistical results are expressed as the mean ± S.E.M. values. The ∗ symbol indicates statistical significance (*p* < 0.05). Student’s *t* test was used to compare pairs of interest.

## Data availability

The data underlying this article will be provided on reasonable request to the corresponding author.

## Conflict of interest

The authors declare that they have no conflicts of interest with the contents of this article.

## References

[1] Ikebe M. (2008). Regulation of the function of mammalian myosin and its conformational change. Biochem. Biophys. Res. Commun..

[2] Mermall V., Post P.L., Mooseker M.S. (1998). Unconventional myosins in cell movement, membrane traffic, and signal transduction. Science.

[3] Bi A.L., Wang Y., Zhang S., Li B.Q., Sun Z.P., Bi H.S. (2015). Myosin II regulates actin rearrangement-related structural synaptic plasticity during conditioned taste aversion memory extinction. Brain Struct. Funct..

[4] Hammer J.A., Wagner W. (2013). Functions of class V myosins in neurons. J. Biol. Chem..

[5] Kneussel M., Wagner W. (2013). Myosin motors at neuronal synapses: drivers of membrane transport and actin dynamics. Nat. Rev. Neurosci..

[6] Rex C.S., Gavin C.F., Rubio M.D., Kramar E.A., Chen L.Y., Jia Y. (2010). Myosin IIb regulates actin dynamics during synaptic plasticity and memory formation. Neuron.

[7] Schenk J., Wilsch-Brauninger M., Calegari F., Huttner W.B. (2009). Myosin II is required for interkinetic nuclear migration of neural progenitors. Proc. Natl. Acad. Sci. U. S. A..

[8] Solecki D.J., Trivedi N., Govek E.E., Kerekes R.A., Gleason S.S., Hatten M.E. (2009). Myosin II motors and F-actin dynamics drive the coordinated movement of the centrosome and soma during CNS glial-guided neuronal migration. Neuron.

[9] Trivedi N., Solecki D.J. (2011). Neuronal migration illuminated: a look under the hood of the living neuron. Cell Adh. Migr..

[10] Ryu J., Liu L., Wong T.P., Wu D.C., Burette A., Weinberg R. (2006). A critical role for myosin IIb in dendritic spine morphology and synaptic function. Neuron.

[11] Gavin C.F., Rubio M.D., Young E., Miller C., Rumbaugh G. (2012). Myosin II motor activity in the lateral amygdala is required for fear memory consolidation. Learn. Mem..

[12] McKenzie J.A., Ridley A.J. (2007). Roles of Rho/ROCK and MLCK in TNF-alpha-induced changes in endothelial morphology and permeability. J. Cell. Physiol..

[13] Malczynska P., Piotrowicz Z., Drabarek D., Langfort J., Chalimoniuk M. (2019). [The role of the brain-derived neurotrophic factor (BDNF) in neurodegenerative processes and in the neuroregeneration mechanisms induced by increased physical activity]. Postepy Biochem..

[14] Notaras M., van den Buuse M. (2019). Brain-derived neurotrophic factor (BDNF): novel insights into regulation and genetic variation. Neuroscientist.

[15] Popova N.K., Naumenko V.S. (2019). Neuronal and behavioral plasticity: the role of serotonin and BDNF systems tandem. Expert Opin. Ther. Targets.

[16] De Vincenti A.P., Rios A.S., Paratcha G., Ledda F. (2019). Mechanisms that modulate and diversify BDNF functions: implications for hippocampal synaptic plasticity. Front. Cell. Neurosci..

[17] Gonzalez M.C., Radiske A., Cammarota M. (2019). On the involvement of BDNF signaling in memory reconsolidation. Front. Cell. Neurosci..

[18] Jin Y., Sun L.H., Yang W., Cui R.J., Xu S.B. (2019). The role of BDNF in the neuroimmune axis regulation of mood disorders. Front. Neurol..

[19] Kermani P., Hempstead B. (2019). BDNF actions in the cardiovascular system: roles in development, adulthood and response to injury. Front. Physiol..

[20] Yoshizaki K., Yamamoto S., Yamada A., Yuasa K., Iwamoto T., Fukumoto E. (2008). Neurotrophic factor neurotrophin-4 regulates ameloblastin expression via full-length TrkB. J. Biol. Chem..

[21] Adelstein R.S., Klee C.B. (1981). Purification and characterization of smooth muscle myosin light chain kinase. J. Biol. Chem..

[22] Klapproth E., Kammerer S., El-Armouche A. (2022). Function and regulation of phosphatase 1 in healthy and diseased heart. Cell Signal..

[23] Ishikawa T., Chijiwa T., Hagiwara M., Mamiya S., Saitoh M., Hidaka H. (1988). ML-9 inhibits the vascular contraction via the inhibition of myosin light chain phosphorylation. Mol. Pharmacol..

[24] Isemura M., Mita T., Satoh K., Narumi K., Motomiya M. (1991). Myosin light chain kinase inhibitors ML-7 and ML-9 inhibit mouse lung carcinoma cell attachment to the fibronectin substratum. Cell Biol. Int. Rep..

[25] Rikitake Y., Kim H.H., Huang Z., Seto M., Yano K., Asano T. (2005). Inhibition of Rho kinase (ROCK) leads to increased cerebral blood flow and stroke protection. Stroke.

[26] Alonso M., Vianna M.R., Izquierdo I., Medina J.H. (2002). Signaling mechanisms mediating BDNF modulation of memory formation *in vivo* in the hippocampus. Cell. Mol. Neurobiol..

[27] Barry S.M., McGinty J.F. (2017). Role of Src family kinases in BDNF-mediated suppression of cocaine-seeking and prevention of cocaine-induced ERK, GluN2A, and GluN2B dephosphorylation in the prelimbic cortex. Neuropsychopharmacology.

[28] Gaidin S.G., Turovskaya M.V., Gavrish M.S., Babaev A.A., Mal'tseva V.N., Blinova E.V. (2020). The selective BDNF overexpression in neurons protects neuroglial networks against OGD and glutamate-induced excitotoxicity. Int. J. Neurosci..

[29] Mao L.M., Geosling R., Penman B., Wang J.Q. (2017). Local substrates of non-receptor tyrosine kinases at synaptic sites in neurons. Sheng Li Xue Bao.

[30] Portugal C.C., Almeida T.O., Socodato R., Relvas J.B. (2021). Src family kinases (SFKs): critical regulators of microglial homeostatic functions and neurodegeneration in Parkinson's and Alzheimer's diseases. FEBS J..

[31] Ye Z., Izadi A., Gurkoff G.G., Rickerl K., Sharp F.R., Ander B.P. (2022). Combined inhibition of Fyn and c-Src protects hippocampal neurons and improves spatial memory via ROCK after traumatic brain injury. J Neurotrauma.

[32] Bhat P., Thorn P. (2009). Myosin 2 maintains an open exocytic fusion pore in secretory epithelial cells. Mol. Biol. Cell.

[33] Brown J., Bridgman P.C. (2003). Role of myosin II in axon outgrowth. J. Histochem. Cytochem..

[34] Even-Ram S., Doyle A.D., Conti M.A., Matsumoto K., Adelstein R.S., Yamada K.M. (2007). Myosin IIA regulates cell motility and actomyosin-microtubule crosstalk. Nat. Cell Biol..

[35] Hodges J.L., Newell-Litwa K., Asmussen H., Vicente-Manzanares M., Horwitz A.R. (2011). Myosin IIb activity and phosphorylation status determines dendritic spine and post-synaptic density morphology. PLoS One.

